# Bilateral pulmonary embolism associated with peripheral blood eosinophilia and positive antiphospholipid antibodies in a patient with cellulitis

**DOI:** 10.1002/ccr3.7313

**Published:** 2023-05-04

**Authors:** Han Naung Tun, May Thu Kyaw, Daryna Chernikova, Mihai Trofenciuc, Syed Haseeb Raza, Mahmoud Abdelnabi

**Affiliations:** ^1^ Larner College of Medicine's UVM Medical Centre University of Vermont Burlington Vermont USA; ^2^ Heart and Vascular Centre Victoria Hospital Yangon Myanmar; ^3^ Cardiology Department City Hospital Kramatorsk Ukraine; ^4^ Institute of Cardiovascular Disease Timisoara and "Vasile Goldis” Western University Timișoara Romania; ^5^ National Institute of Cardiovascular Diseases Karachi Pakistan; ^6^ Internal Medicine Department Texas Tech University Health Science Center Lubbock Texas USA

**Keywords:** anticoagulants, antiphospholipid antibodies, cellulitis, eosinophilia, thrombosis

## Abstract

**Key Clinical Message:**

This report described the pathophysiology, diagnostic workup, and management of thrombosis possibly associated with peripheral blood eosinophilia and transient positive antiphospholipid antibodies in the setting of cellulitis.

**Abstract:**

Peripheral blood eosinophilia is a risk factor for thrombosis and the presence of other prothrombotic factors such as antiphospholipid antibodies can potentiate that risk. The authors present a case of acute pulmonary embolism which developed at the peak of eosinophilia, later found to have transient positive antiphospholipid antibodies in a male patient with right lower limb cellulitis and a history of intravenous drug abuse. This report illustrates the pathophysiology, diagnosis workup, and therapeutic options of thrombosis possibly associated with peripheral blood eosinophilia and positive antiphospholipid antibodies, which include anticoagulants, corticosteroids, and immunosuppressants. Clinicians should be aware of this possible association which may guide the choice and duration of anticoagulants. Although direct oral anticoagulants are effective anticoagulants in various thromboembolic events, studies showed unfavorable outcomes for their use in antiphospholipid syndrome.

## 1 BACKGROUND

Tissue and/or blood eosinophilia are reported in several conditions including infectious, inflammatory, allergic, and neoplastic disorders. Eosinophilia‐related clinical manifestations are diverse and include tissue fibrosis or thrombosis. Peripheral blood eosinophilia (PBE), even if transient, can be prothrombogenic.^1^ Antiphospholipid antibodies (APLAs) are associated also with an increased risk of thrombosis.^2^, ^3^ Several bacterial and viral infections were associated with transient APLAs such as bacterial sepsis, syphilis, human immunodeficiency virus (HIV), Epstein–Barr virus (EBV), cytomegalovirus, severe acute respiratory syndrome coronavirus 2 (SARS‐CoV‐2).^4^, ^5^, ^6^ Cases of venous thromboembolism (VTE) occurring in the settings of eosinophil‐related diseases have been rarely reported. Herein, the authors present a case of VTE with pulmonary embolism (PE) which developed at the peak of eosinophilia, later found to have transient positive antiphospholipid antibodies (APLAs) in a male patient with right lower limb cellulitis and a history of intravenous drug abuse.

## 2 CASE PRESENTATION

**FIGURE 1:**
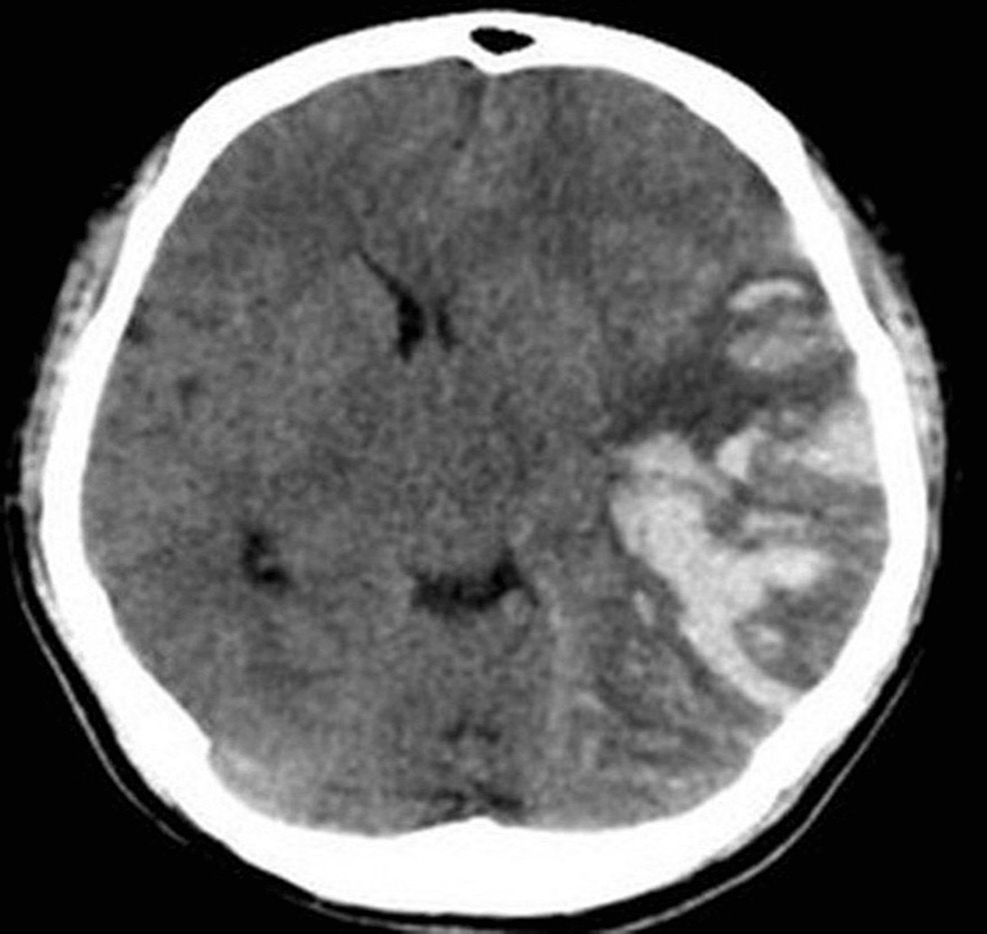
CT brain (axial view) showing acute hemorrhagic cerebral infarction at the left temporoparietal region with midline shift and mass effect.

**FIGURE 2:**
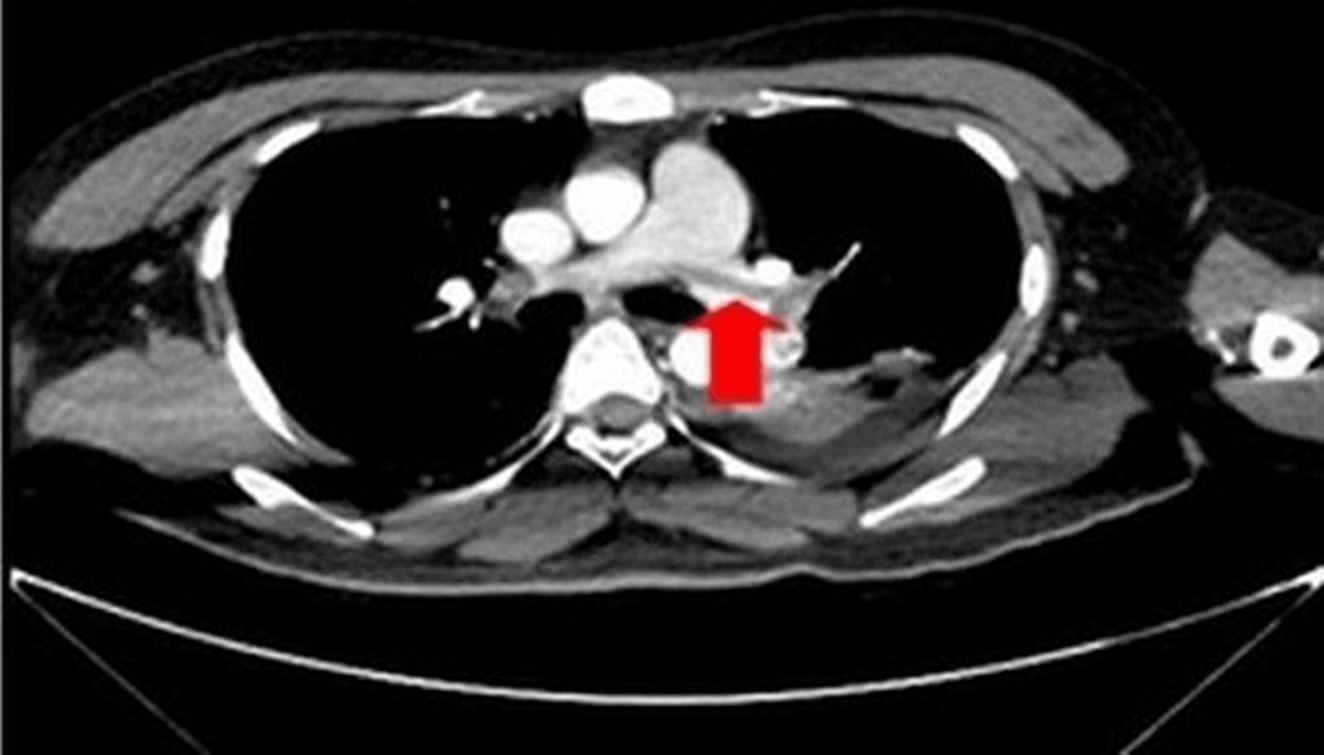
CTPA showing large intraluminal thrombus in both right and left main pulmonary arteries and nearly complete obstruction of both lower lobes' pulmonary arteries highlighted by a red arrow.

A 26‐year‐old male patient with a history of intravenous drug abuse presented with an ulcer at the right upper thigh for 3 days which was diagnosed as lower limb cellulitis with impending abscess by computed tomography (CT) scan and treated with surgical wound debridement and broad‐spectrum antibiotics. Preoperative complete blood count (CBC) showed a hemoglobin (Hb) level of 15.6 g/dL, total leukocyte count of 14.69 × 10^9^/L (normal: 4.5–11), platelet count of 107 × 10^9^/L, with a differential count of polymorphs 73%, lymphocytes 12%, monocytes 7.4%, and eosinophils 6.2% (absolute neutrophil count: 10.78 × 10^9^/L and absolute eosinophil count: 0.91 × 10^9^/L). C‐reactive protein was elevated at 84.49 mg/L (normal range 0.3–1.0) while renal and liver function tests were within normal range and tissue culture from the ulcer and blood cultures were negative. First day postoperatively, he developed a new onset of neurological symptoms in the form of slurred speech and weakness in all four limbs with a Glasgow Coma Scale (GCS) of 11/15 (Eye 3, Verbal 4, Motor 4). CT brain showed acute hemorrhagic cerebral infarction at the left temporoparietal region with midline shift and mass effect. (Figure 1). Accordingly, decompressive craniotomy with partial temporal lobectomy (left side) was done. Intraoperatively, arteriovenous malformation with hemorrhage was noted which was confirmed later by histopathology. During the craniotomy, 2 units of fresh frozen plasma, 2 units of fresh whole blood, and 2 units of platelet‐rich plasma were given and another 2 units of PRP were given on the immediate postoperative day due to his low platelet count. Serial monitoring showed progressive neutrophilia, eosinophilia, and thrombocytopenia. (Table 1). Stool examination for ova and parasites was negative. However, on the 5th postoperative day, at the peak of eosinophilia (14.03 × 10^9^/L, 44.3%), he developed sudden onset of dyspnea with reduced oxygen saturation (SaO_2_ 67%). An electrocardiogram (ECG) showed sinus tachycardia with a heart rate of 120 beats per minute. Echocardiography showed a dilated right ventricle (RV) with RV strain (TAPSE = 14 mm) with functional moderate tricuspid regurgitation. Arterial blood gases (ABG) showed type I respiratory failure. His Wells score for PE was 6 points with an elevated D‐dimer level (>10 mg/L). CT pulmonary angiogram (CTPA) showed large intraluminal thrombi in both his right and left main pulmonary arteries with nearly complete obstruction of both lower lobes' pulmonary arteries (Figure 2). His Wells' score for deep vein thrombosis (DVT) was 1 point. Color Doppler ultrasound of bilateral lower limb veins revealed thrombosis in the right popliteal vein. Immunologic workup included antinuclear antibody (ANA), antidouble stranded DNA (anti dsDNA), histone antibodies, and screening for paroxysmal nocturnal hemoglobinuria (PNH) were all negative. Thrombophilia screen showed positive β2 glycoprotein (β2GP) IgM (25.63 RU/mL) and detectable lupus anticoagulant (LA) with negative anticardiolipin (aCL) antibodies. Additionally, p‐ANCA was weakly positive. His coagulation profile was within the normal range. A treatment strategy based on subcutaneous low‐molecular‐weight heparin (LMWH), enoxaparin (1 mg/kg twice daily) was started and after 5 days, changed to apixaban 2.5 mg twice daily which was stopped after positive LA and subcutaneous LMWH was restarted, enoxaparin (1 mg/kg twice daily). In addition, he was treated with a thrombopoietin receptor agonist, eltrombopag 50 mg twice daily, and a fusion protein analog of thrombopoietin, SC romiplostim once a week, plus low‐dose steroid, dexamethasone 2 mg once daily which showed improvement on platelet count and eosinophil count. His final predischarge platelet level was 126 × 10^9^/L and his eosinophil count was 2.25 × 10^9^/L (i.e., a reduction of 17.2%). He was discharged on oral dexamethasone for 10 days. Subcutaneous LMWH was changed to warfarin titrated to reach a target international normalized ratio (INR) of 2.5–3.5 for 6 months. At a 6‐month follow‐up, his laboratory workup showed no recurrence of eosinophilia with normal platelet count and negative APS antibodies.

**TABLE 1:** Serial monitoring of complete blood count.

CBC	Day 1	Day 2	Day 3	Day 5	Day 6	Day 7	Day 10	Day 18	Day 21
Total white cell count (× 10^9^/L)	13.26	18.54	29.28	37.00	27.29	31.68	29.59	11.85	13.11
Neutrophils (× 10^9^/L)	10.07 75.8%	8.57 46.2%	12.94 44.2%	17.27 46.6%	14.37 52.7%	12.97 40.8%	15.03 50.9%	5.45 46%	7.93 60.5%
Lymphocytes (× 10^9^/L)	1.24 9.4%	2.16 11.7%	2.63 9.0%	2.29 6.2%	1.70 6.2%	1.92 6.1%	3.05 10.3%	1.96 16.5%	1.52 11.6%
Monocytes (× 10^9^/L)	1.14 8.6%	1.15 6.2%	1.81 6.2%	2.31 6.3%	1.75 6.4%	2.53 8.0%	2.46 8.3%	1.50 12.7	1.25 9.5%
Eosinophils (× 10^9^/L)	0.79 6.0	6.58 35.5%	11.71 40%	14.95 40.4%	9.38 34.4%	14.03 44.3%	8.86 29.9%	2.83 23.9%	2.25 17.2%
Red blood cell count (million/mm^3^)	3.74	3.37	3.41	3.29	3.71	3.66	4.37	3.52	3.73
Hb (g/dL)	10	8.9	9.2	8.8	10	9.8	11.5	9.0	9.6
Platelets (× 10^9^/L)	42	57	28	46	49	47	89	98	126

## 3 DISCUSSION

Hypereosinophilic syndromes (HES) are defined by persistent eosinophilia >1500 eosinophils/μL with end organ dysfunction, in the absence of secondary causes of eosinophilia. HES is more common in middle‐aged men, characterized by both pulmonary (in 40%) and extrapulmonary involvement. The severity of organ involvement is governed by two factors: first, the level of eosinophilia (high effector cell burden), and second, the duration of eosinophilia (persistent).^1^ Thus, this case fulfilled HES criteria based on peripheral eosinophilia, complicated by venous thromboembolism (VTE) and PE, which occurred on the day of maximum PBE. Although the precise mechanism of eosinophil‐induced hypercoagulability is still unclear, contributing factors probably include initiation of the clotting cascade by expression of tissue factor, endothelial injury by major basic protein (MBP), stimulation of thrombus formation by binding to heparin and neutralizing its anticoagulating effects by eosinophilic cationic protein.^7^, ^8^ Data regarding the risk of thrombosis due to eosinophilia is limited to case reports and a small cohort study that showed that within a 29‐month follow‐up, 24% of the included patients, had one or more thrombotic events.^9^ Antiphospholipid syndrome (APS) is associated with an increased risk of thrombosis which is medicated by impairment of fibrinolysis, reduction in protein C activity, procoagulant effects on platelets and endothelial cells, and induction of vascular endothelial growth factor (VEGF).^2^, ^3^, ^10^ Diagnosis of APS is done by revised Sapporo classification criteria which include clinical criteria (vascular thrombosis and pregnancy morbidity) and laboratory criteria: LA, aCL, and anti‐β2 glycoprotein I antibodies.^11^ Some major clinical manifestations are not included in the revised Sapporo classification criteria: thrombocytopenia, hemolytic anemia, and renal, cardiac, dermatological, and neurological involvement. Thus, this case met one clinical criterion (vascular thrombosis) and two laboratory criteria (positive Anti‐β2‐glycoprotein IgM and positive LA) although antibodies should be repeated after 12 weeks apart. The standard treatment for thrombotic APS is initial anticoagulation with heparin followed by warfarin^12^, ^13^ Previous reports have linked bacterial infections with an acute onset of APS.^14^ Martin et al, reported a case report of catastrophic APS in a community‐acquired methicillin‐resistant Staphylococcus aureus infection.^15^ History of drug use, upper thigh ulcer, and cellulitis in our case might suggest an infectious etiology of APS yet he was started on antibiotics therefore, cultures came back negative. Direct oral anticoagulants (DOACs) are not effective alternatives for heparin or warfarin in APS.^15^ ASTRO‐APS trial reported unanticipated excessive risk of arterial events during the first months in the apixaban group when compared to warfarin.^16^, ^17^ Moreover, studies showed that recurrent thrombosis occurred in APS despite adequate anticoagulation with DOACs. Therefore, in this case, once the diagnosis of APS was considered, a switch from apixaban to LMWH was done. What is also worth mentioning, is that this case did not fulfill the criteria for eosinophilic granulomatosis with polyangiitis (EGPA)^18^ as a case of eosinophilia and APLs antibodies as a cause of his peculiar presentation.

## 4 CONCLUSION

PBE by itself is thrombogenic and the presence of positive APLAs potentiates this. This combination, although rare, can occur and may result in significant morbidity and mortality. Despite the limited data, our case highlights the coexistence of eosinophilia and positive APLAs which may be an important factor for different thromboses, that warrant timely recognition and appropriate management. Moreover, today, although DOACs are widely used with encouraging outcomes in different settings of atherosclerotic vascular diseases, DOACs are not recommended in APS patients especially if they have other prothrombotic factors.

## AUTHOR CONTRIBUTIONS

**Han Naung Tun:** Writing – original draft; writing – review and editing. **May Thu Kyaw:** Writing – original draft; writing – review and editing. **Daryna Chernikova:** Writing – original draft; writing – review and editing. **Mihai Trofenciuc:** Writing – original draft; writing – review and editing. **Syed Haseeb Raza:** Writing – original draft; writing – review and editing. **Mahmoud Abdelnabi:** Writing – original draft; writing – review and editing.

## FUNDING INFORMATION

None.

## CONSENT

Patient consent has been signed and collected in accordance with the journals patient consent policy. The authors have obtained written informed consent from the patients medical power of attorney to publish his medical history/course and case details in accordance with the journals patient consent policy case.

## Data Availability

The data are available for sharing.
